# Letter from the Editor in Chief

**DOI:** 10.19102/icrm.2024.15046

**Published:** 2024-04-15

**Authors:** Moussa Mansour


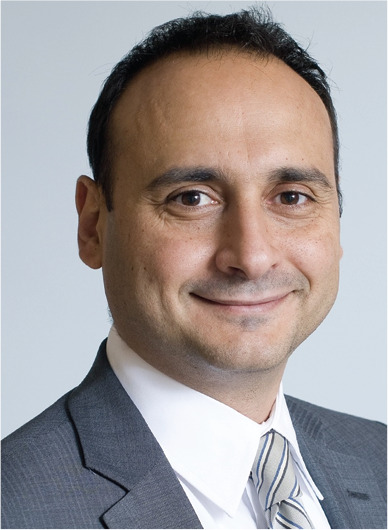


Dear readers,

The annual scientific meeting of the European Heart Rhythm Association was held earlier this month in Berlin. Some of the most significant sessions of the meeting were the late-breaking clinical trial sessions. Although all the studies that were presented were important, I will highlight two presentations related to the ablation of ventricular tachycardia (VT), which I believe will gain significant attention in the next few years.

The first study was the Safety and Effectiveness of Ultra-low-temperature Cryoablation of Monomorphic Ventricular Tachycardia in Patients with Ischemic and Non-ischemic Cardiomyopathies (Cryocure-VT) trial.^1^ It was presented by Dr. Atul Verma and described the safety and efficacy of ultra-low-temperature cryoablation for the treatment of ventricular tachycardia (VT). Sixty-four patients with cardiomyopathy and VT were included. Clinical VT was acutely eliminated in 94% of the patients, and, at follow-up, 81% of patients were free of implantable cardioverter-defibrillator (ICD) shocks. Serious adverse events occurred in 6% of the participants and included pericardial effusions and cardiogenic shock.

The second study of note was the Impact of Preventive Substrate Catheter Ablation on Implantable Cardioverter-defibrillator Interventions in Patients with Ischemic Cardiomyopathy and Infarct-related Coronary Chronic Total Occlusion (PREVENTIVE VT) trial presented by Dr. David Žižek.^2^ This was a randomized controlled trial comparing preventive ablation plus ICD versus ICD only in patients with chronic total occlusion of a coronary artery who also had an ejection fraction of <40% and a primary indication. The primary endpoint was time to ICD therapy or VT-related hospitalization. Sixty patients were enrolled and randomized between the two groups. At follow-up, the primary composite outcome occurred in 16.7% and 43.3% of patients, respectively, suggesting that preventive ablation of chronic total occlusion substrate at the time of primary prevention ICD implantation reduces the risk of ICD therapy and VT-related hospitalization.

Although each of these studies included a small number of patients, both are important because they expand our knowledge of VT ablation. The current literature on VT ablation is lacking in the areas of novel technology and indications. Radiofrequency energy has significant limitations for VT ablation, with mainly lesion depth limiting the achievement of transmural lesions. Similarly, the indications of VT ablation are poorly defined. The two studies discussed above will hopefully set the foundation or facilitate larger multicenter studies for VT ablation to help address the unmet needs.


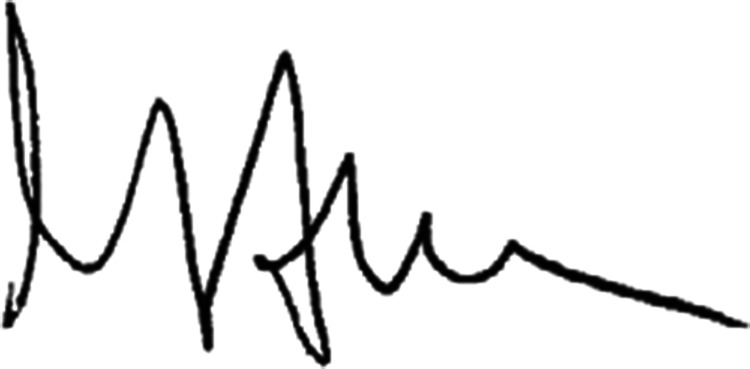


Best regards,

Moussa Mansour, md, fhrs, facc

Editor in Chief

*The Journal of Innovations in Cardiac Rhythm Management*

MMansour@InnovationsInCRM.com

Director, Atrial Fibrillation Program

Jeremy Ruskin and Dan Starks Endowed Chair in Cardiology

Massachusetts General Hospital

Boston, MA 02114
